# Examining the Potential Scaling Law in Urban PM2.5 Pollution Risks along with the Nationwide Air Environmental Effort in China

**DOI:** 10.3390/ijerph19084460

**Published:** 2022-04-07

**Authors:** Lei Yao, Wentian Xu, Ying Xu, Shuo Sun

**Affiliations:** 1 College of Geography and Environment, Shandong Normal University, Jinan 250014, China; alex_yaolei@126.com (L.Y.); xuwentian0130@outlook.com (W.X.); sdnu_ss@163.com (S.S.); 2 School of Civil Engineering, Shandong Jiaotong University, Jinan 250023, China

**Keywords:** air pollution risk, sublinear relationship, urbanization, zoning analysis, scale-adjusted metropolitan indicator

## Abstract

Urban scaling law provides a quantitative understanding of the fundamental nonlinear properties of how cities work. Addressing this, this study intended to examine the potential scaling law that may lie in urban air pollution. With ground-monitored PM2.5 data and statistical socioeconomic factors in 265 Chinese cities (2015–2019), a targeted analysis, based on the scaling power-law model and scale-adjusted metropolitan indicator (SAMI) was conducted. The main findings of this study were summarized as follows: (1) A significant sublinear scaling relationship between PM2.5 and urban population size indicated that air quality degradation significantly lagged behind urban growth, affirming the remarkable effectiveness of national efforts on atmospheric environment improvement. (2) SAMI analysis expressed the relative conflict risk between PM2.5 pollution and urbanization and showed significant spatial cluster characteristics. Cities in central China showed higher potential risk than other regions, and there was a clear southward tendency for the city clusters with increasing SAMIs during the study period. (3) During the study period, urbanization was not the reason affecting the human-land conflict in terms of air pollution. This study is significant in that it marked the first innovative incorporation of the scaling law model into an urban environmental risk study. It also offered a new perspective from which to reframe the urban PM2.5 pollution risk, along with the nationwide air environmental effort in China, which will benefit future research on multi-types of urban environmental issues.

## 1. Introduction

The process of urbanization is one of the most significant manifestations of human civilization reshaping the Earth’s surface, accompanied by the continuous migration and growth of the population [1]. Urban demographic growth produces ever-greater demands on services for better living, externalizing as general affluence in multiple urban indicators, such as economy level, individual income, infrastructure coverage, and traffic volume [2,3]. However, today’s cities vary greatly in enormous and changing scales (or sizes), from small cities with just a few people to megacities with tens of millions of inhabitants, leading to a wide range of urban indicators in the form of complexity and heterogeneity [4,5]. This objective reality thus causes difficulties in recognizing the coherent linkages between these urban indicators and the accompanying urbanization process across different cities.

**Figure 1:**
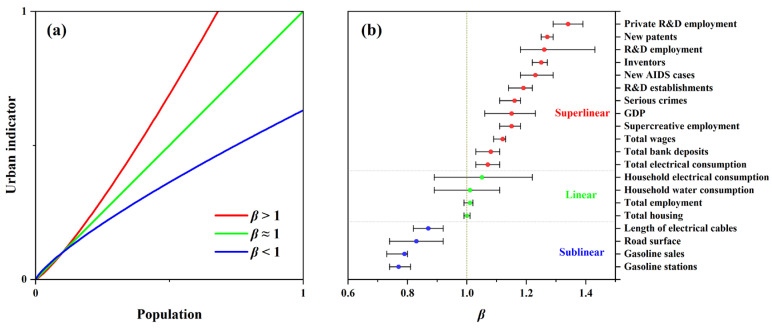
Schematic of three categories of urban scaling exponent: (**a**) superlinear, linear, and sublinear, and (**b**) the average scaling exponents (*β*) with ±95% confidence interval (CI) for different urban indicators of the cities in China, EU, and USA, summarized by Bettencourt, Lobo [8]. R&D: research and development.

Benefitting from increased data availability in many urban systems, urbanologists began to experiment with a simplified analytical framework, i.e., urban scaling law, to generalize the statistical patterns of urban indicators dependent on city scales [4,6,7]. The scaling model was proposed based on dense empirical studies documenting that most urban properties vary continuously with urban scale, and can be well quantified by nonlinear power-law relations (*Y*~*Y*_0_·*N^β^*). *Y* indicates a specific urban indicator (e.g., road network length and employment), *Y*_0_ is the normalization constant, *N* is the population size (usually as the measure of city scale), and *β* indicates the general scaling rule across an urban system. Bettencourt and Lobo [8] systematically reviewed previous relevant studies and argued that the index of *β* acted as a robust scaling exponent for a wide variety of urban indicators across different countries or time periods. Then, the range of *β* fell into three categories with a taxonomic universality clustering around similar values, as: *β* > 1 (a superlinear relationship, signifying increasing returns to population size (scale), such as the growth of gross domestic product (GDP) being faster than demographic growth), *β* ≈ 1 (a linear relationship, associating with individual human needs which can be treated as a standard per capita measurement), and *β* < 1 (a sublinear relationship, expressing economies of scale usually associated with urban infrastructure) (Figure 1). With this, the unified scaling model thus facilitates determination of the evolution of urban indicators, along with the urbanization process, benefitting future urban policymaking [7,9,10,11]. However, most current urban scaling studies emphasized the endogenous indicators of cities, e.g., socioeconomic activities or urban infrastructures [8,12,13,14]. Thus, a question could be posed: could the urban scaling law model be effectively extended to characterize the exogenous factors of urban systems such as urban environmental degradation?

The process of urbanization, while bringing about a great accumulation of population, wealth, architecture, technology, and other resources, will also exert negative externalities on itself and surrounding regions and induce serious eco-environmental crises [1,15]. Among the multiple types of environmental degradations, ambient air pollution has aroused widespread concerns recently due to its inescapability and high risk of morbidity and mortality [16,17,18]. The primary sources of air pollution are fossil combustion, diesel vehicle usage, and household and industrial activity, etc., all of which are closely related to urbanization and industrialization processes [19]. For this reason, recent academic discussions on air pollution have been carried out in the context of urbanization, covering topics such as spatiotemporal characteristics [20,21,22], driving factors [23,24,25], and associated risk stress [26,27,28]. On the whole, findings of these studies affirmed the pivotal contribution of urban development on local, regional, or even global atmospheric environment deterioration, especially in developing nations or regions undergoing dramatic urbanization or industrialization (i.e., where the air pollution condition enhanced progressively with their development) [29,30]. Most relevant studies adopted absolute concentration of pollutants to characterize the pollution status for specific cities or city-to-city comparison [31], but they failed to consider that the differences in urban scale may be a potential confounding factor during the assessment of air pollution status. For example, two Chinese cities, Beijing and Nanchong, showed similar annual fine particulate matter (PM2.5) pollution levels in 2019 (42 μg/m^3^, http://www.moc.cma.gov.cn accessed on 12 May 2020), but with huge exposure demographic differences of >20 and 6.43 million residents, respectively [32]. Thus, relying solely on pollutant concentration is not sufficient to express the relative risks to which different cities are exposed. Moreover, recent studies have emphasized the solid nonlinear relationship between the air pollutant and urban (population) scale across different cities [33,34]. Thus, whether urban environmental risks follow urban scaling law becomes a topic worthy of academic discussion.

Echoing the above assumptions, this study intended to examine the effectiveness of urban scaling laws in the wider context of urbanization. To the best of our knowledge, we made the first attempt to explore urban air pollution issues and the potential scaling law characteristics that they may imply. PM2.5 was considered as the typical air pollutant for this study due to its higher topicality and attention-grabbing nature in both academic and public society [16,17]. The specific research objectives were as follows: (1) verifying the effectiveness of an urban scaling law on urban PM2.5 pollution in Chinese cities, along with nationwide air pollution regulation (2015–2019); (2) characterizing the spatiotemporal features of the relative PM2.5 pollution risk by introducing the Scale-Adjusted Metropolitan Indicator (SAMI) based on a scaling model; (3) examining whether the urbanization process contributed to variation in the relative PM2.5 pollution risk. Then, in-depth and extended discussions based on the main findings were conducted to clarify the potential theoretical and practical insights into the recognition of urban PM2.5 pollution risk.

## 2. Materials and Methods

### 2.1. Study Area

**Figure 2:**
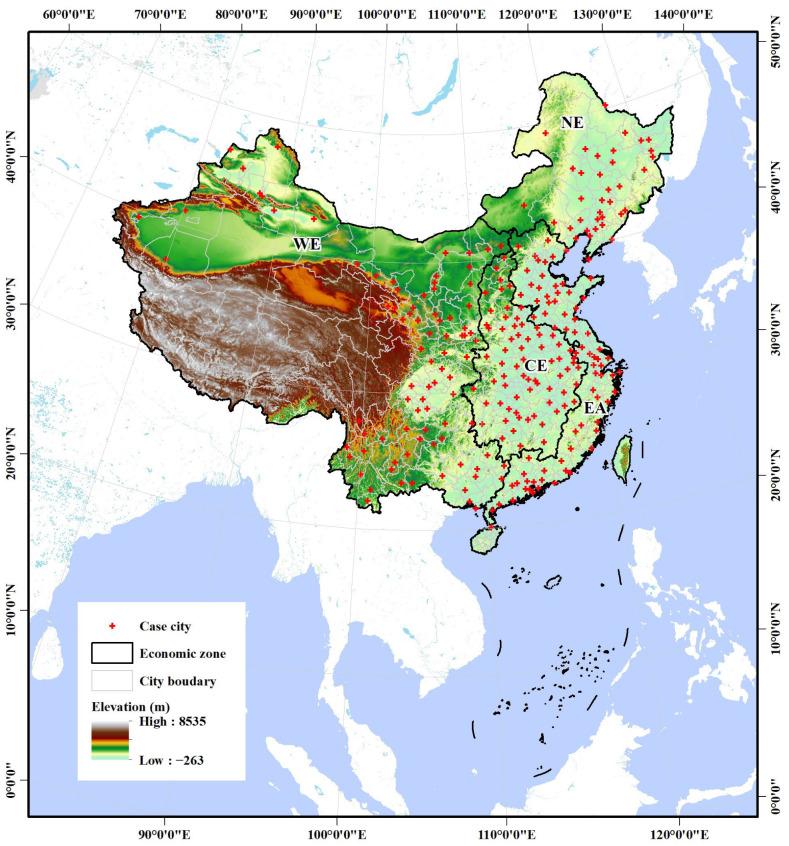
The case cities in China and related economic zones of this study. NE: northeast China; EA: east China; CE: central China; WE: west China.

In this study, we focused our attention on cities in China, which are experiencing remarkable urban transformation benefiting from the Reform and Opening-up policy [15,35]. However, the consequent deterioration of air quality triggered by urbanization and industrialization processes has aroused widespread concern from all sectors of society, and PM2.5 has quickly become a widely known buzzword in public opinion [16,36]. Since 2013, therefore, the Chinese government has advocated a series of atmospheric environment-related policies in an effort to improve the severe nationwide air pollution crisis [37,38]. Thanks to this, local governments have built systematic monitoring systems for PM2.5 and other air pollutants across the country (Figure 2). This, coupled with China’s huge urban population base (in terms of both development momentum and risk exposure potential) [39,40] and significant regional differences among cities (in terms of climate, socio-economic, and environmental pollution risk, etc.) [41,42,43] provided us adequate environmental monitoring data and the diversified caseload needed in this study.

### 2.2. Data Preparations

The nationwide records of the hourly PM2.5 concentration data started in early 2014 by the ground-level air quality monitoring stations in Chinese cities, provided by the urban air quality publishing platform of China National Environment Monitoring Center (http://www.moc.cma.gov.cn accessed on 12 May 2020). To ensure the integrity and comparability of data sources, we initially excluded cities with irreparable data deficiencies, and the time span of 2015–2019 was determined to maximize the integrity of recorded data for most cities. Eventually, this study selected 265 cities across the northeast (NE, with 36 cities), east (EA, 74), central (CE, 69), and west (WE, 86) economic zones of mainland China for empirical case study (Figure 2), involving municipalities, prefecture-level cities, and autonomous prefectures. The continuous recorded hourly PM2.5 documentations of the 265 case cities during 2015–2019 were downloaded, then were (average) calculated with all the time nodes of all the monitoring stations to obtain the annual PM2.5 concentration for each city [33].

According to the urban scaling law model [6,11,40], the permanent resident population (POP, 10,000 person) was selected as the basic indicator to quantify each case city’s size. Moreover, plenty of relevant studies have documented that urbanization processes pose an incontestable impact on PM2.5 pollution worldwide [29,44,45]. Thus, we tried to explore whether urbanization was still valid for intervening in the scaling performance assigned with PM2.5 pollution. Urban built-up area (BA, km^2^) as well as GDP (100 million RMB), indicating the scale of spatial and economic urbanization respectively [46,47], were chosen for further analysis and discussion in this study. The annual values of all three indicators of the case cities (during 2015–2019, consistent with that of the PM2.5 data) were acquired from the China Urban Statistical Yearbook [32].

### 2.3. Analysis Implementations

#### 2.3.1. Scaling Law Analysis

In this study, we treated the urban PM2.5 pollution as the consequence of urbanization, similar to other types of urban metrics (e.g., infrastructure construction, economic development, production and living consumption, etc.) [44,48,49]. According to the urban scaling law model mentioned in our introduction, the scaling law model on PM2.5 pollution could be localized based on our research targets in the following form:

(1) $$\mathrm{PM}2.5\left(t\right)=\alpha \cdot \mathrm{POP}{\left(t\right)}^{\beta }$$

where PM2.5(*t*) refers to the annual PM2.5 concentration of case cities at time *t*. With this, the relative change in the per capita quantity (PM2.5(*t*)/POP) with each fractional increase in population size (ΔPOP/POP) depended only on *β* instead of the initial city scale, POP(*t*). Then, for the purpose of keeping the model as simple as possible [9,40], the linear ordinary least squares regression was applied to derive the scaling exponent *β* through logarithmic transformation of Equation (1), as:

(2) $$\mathrm{LOG}\left(\mathrm{PM}2.5\left(t\right)\right)=\beta \cdot \mathrm{LOG}\left(\mathrm{POP}\left(t\right)\right)+\mathrm{LOG}\left(\alpha \right)+\xi$$

where *β* can be treated as the slope of the linear model and *ξ* is a normally distributed error with zero mean in a logarithmic relationship. The regression model was examined with a given significance level of 0.05. The value of *β*, obtained from the regression model (passing significance test) was used to determine the scaling characteristics by referring to Figure 1.

However, the scaling exponent *β* was analogous to an overview on the average (power-law scaling) behavior of urban indicators’ response to the demographic size of cities (Figure 1). Therefore, a dimensionless indicator with relative value was developed to further eliminate the impact of population size on urban indicators, according to the study by Bettencourt, Lobo [50], as a Scale-Adjusted Metropolitan Indicator (SAMI):

(3) $$\mathrm{SAMI}=\mathrm{LOG}\frac{Y_{i}}{Y\left(N_{i}\right)}=\mathrm{LOG}\frac{Y_{i}}{Y_{0}\cdot N^{\beta }{}_{i}}$$

where *Y_i_* is the observed value of the urban indicator for specific city *i*. Accordingly, the above scaling information can be substituted into Equation (3) to obtain the SAMI representing the urban PM2.5 pollution risk, as followed:

(4) $$\mathrm{SAMI}=\mathrm{LOG}\frac{\mathrm{PM}2.5{\left(t\right)}_{i}}{\mathrm{PM}2.5\left(\mathrm{POP}{\left(t\right)}_{i}\right)}=\mathrm{LOG}\frac{\mathrm{PM}2.5{\left(t\right)}_{i}}{\alpha \cdot \mathrm{POP}{\left(t\right)}^{\beta }{}_{i}}$$

where PM2.5(*t*)*_i_* is the observed value of annual PM2.5 concentration for the case city *i* in time *t*, while PM2.5(POP(*t*)*_i_*) is the corresponding projected value based on Equation (1). As an index independent of city size, SAMI was able to capture the true local flavor assigned to specific urban characteristics (PM2.5 concentration in this study) to specified times and places, which allowed direct city-to-city performance comparison and provided objective and meaningful rankings across the urban system compared to that of traditional per capita or average indices [11,50]. In this study, the value of SAMI was used to quantify the deviation between the actual PM2.5 pollution level and the expected one in a certain population size according to the urban scaling law (Equation (1)). SAMI < 0 indicated that the actual PM2.5 pollution was weaker than expected with lower urban pollution potential, while SAMI > 0 indicated the opposite performance.

#### 2.3.2. Auxiliary Analysis

With the results of the scaling law analysis, further auxiliary analyses were conducted in this study to deepen the insight of the scaling characteristics of the urban PM2.5 pollution risk in China. First, geospatial models, i.e., Hotspot (Getis-Ord Gi*) and Standard deviational ellipse distributing (SDE) analyses were adopted to identify the statistically significant spatial clusters of high (hotspot) and low values (coldspot) of SAMIs and their spatial tendencies across time and space [47,51,52]. Then, four economic districts, i.e., NE, EA, CE, and WE (Figure 2) were introduced for additional zonal statistics and comparisons with SAMIs using one-way ANOVA and linear trend analysis, in order to capture the dynamic pollution risk characteristics that may exist on the reginal scale [53]. Lastly, an additional regression analysis was conducted between three urbanization indicators (i.e., POP, BA, and GDP) and the varying SAMI (ΔSAMI, gap between 2015 and 2019), to verify whether there was a significant association between the air pollution risk evolution and urbanization process.

In summary, scaling law (including *β* and SAMI), ANOVA, and regression analyses were conducted in SPSS software (Version 23, IBM, Armonk, NY, USA) and plotted in Origin Pro (Version 2021b, OriginLab Corporation, Northampton, MA, USA) and GraphPad Prism software (Version 8.4, GraphPad Software, San Diego, CA, USA), while the geospatial analysis (including hotspot and SDE analysis) and mapping were accomplished in ArcGIS software (Version 10.8, ESRI, Redlands, CA, USA).

## 3. Results

**Figure 3:**
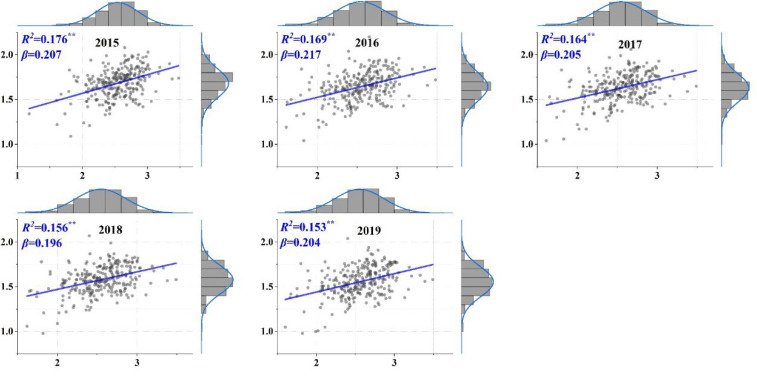
The fitted urban scaling law for logarithmic (LOG) population (POP) and annual PM2.5 concentration (PM2.5) data using linear ordinary least squares regression (Equation (2)) during 2015–2019. The straight line refers to the expected linear relationship between LOG (POP, *x*-axis) and LOG (PM2.5, *y*-axis) in the case cities. *β*: the scaling exponent, **: *p* < 0.01.

The linear fitted results between the logarithmic POP and PM2.5 data showed that the two indicators conformed to the urban scaling law during 2015–2019, even with lower but significant *R*^2^ (0.153–0.176, *p* < 0.01, Figure 3). The scaling exponents (*β*) of PM2.5 ranged from 0.196 (2018) to 0.207 (2015), which was well below the empirical threshold of 1, as shown in Figure 1. This indicated that the scaling feature of PM2.5 pollution showed a significant sublinear pattern during the study period, signifying that growth in urban population size corresponded to lower expectations of air pollution exacerbation from a global perspective.

**Figure 4:**
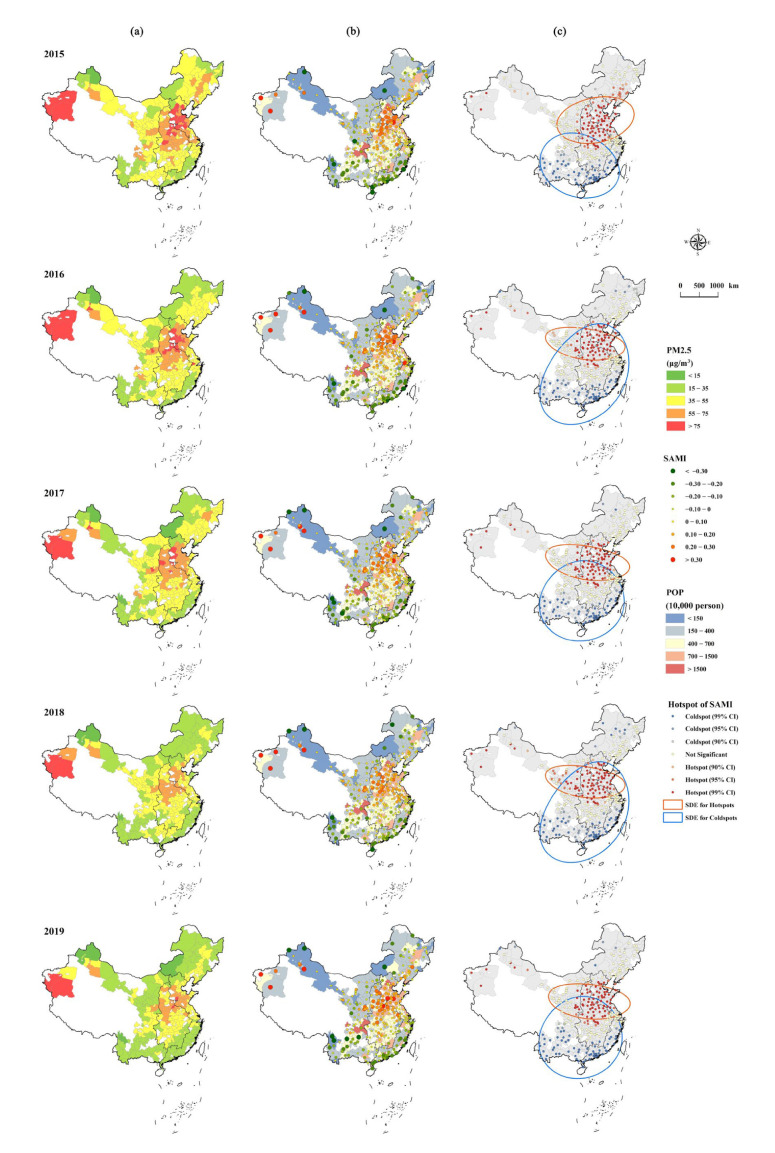
The spatial details of the (**a**) annual PM2.5 concentration (PM2.5), (**b**) Scale-Adjusted Metropolitan Indicators (SAMI) & POP, and (**c**) Hotspot and corresponding standard deviational ellipse (SDE) analysis results of SAMI for the case cities during 2015–2019.

**Figure 5:**
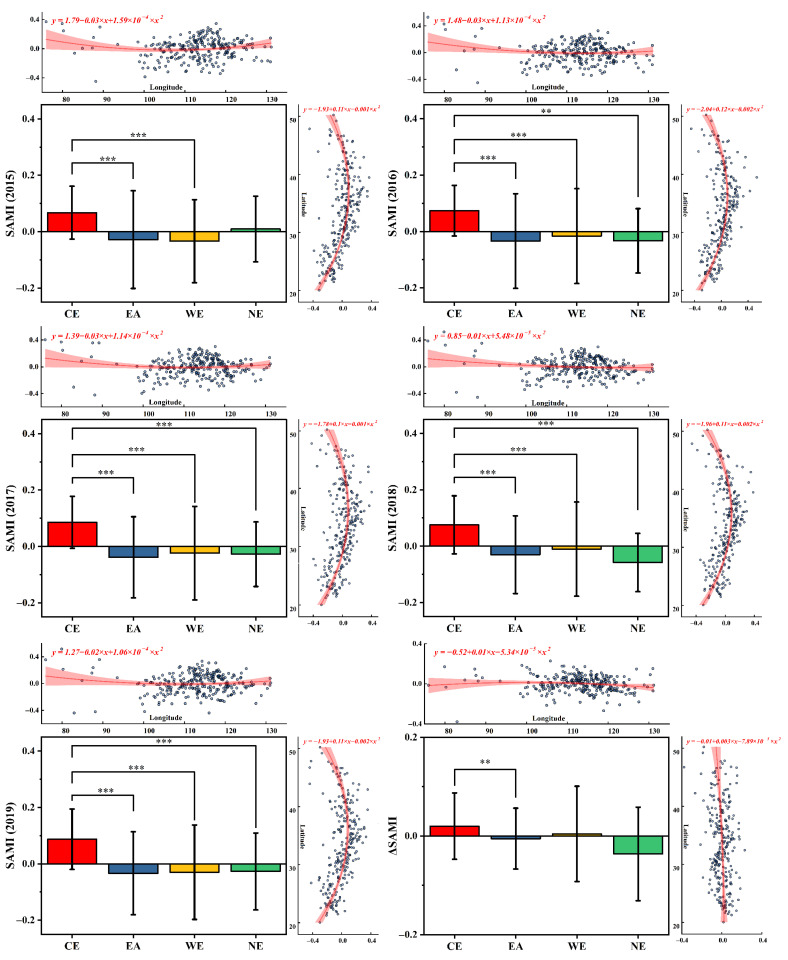
The zonal comparisons of SAMIs (during 2015–2019) and the changing SAMI (ΔSAMI, the SAMI values in 2019 minus that in 2015) between CE, EA, WE, and NE using one-way ANOVA analysis (average value ± standard deviation, Turkey method, ***: *p* < 0.001, **: *p* < 0.01) [33]. The attached scatter plots and their fitted (quadratic polynomial) lines illustrate the general trend of SAMIs/ΔSAMI with longitude (°, horizontal axis) and latitude (°, vertical axis) cross China.

When the focus shifted to the relative difference among individuals, SAMIs of each case city showed significant spatial heterogeneity across the whole study region, as shown in Figure 4. Generally, the spatial distribution of SAMIs was highly compatible with that of PM2.5 in terms of perception. For all of 2015–2019, cities with higher SAMIs were mainly distributed in the North China Plain and the western border regions, where most of the cities also bore higher PM2.5 pollution risks (>55 μg/m^3^). In contrast, lower-value cities were mainly located in the southeast coastal and northern border regions. These spatial variations were further verified by hotspot analysis (Figure 4c). From a zoning perspective, as shown in Figure 5, cities in CE unsurprisingly showed the highest average value of SAMIs across all years, varying from 0.07 (2015, 2016) to 0.09 (2017, 2019), while cities in EA, WE, and NE all showed significantly lower average SAMIs (*p* < 0.01) but larger standard deviations from CE.

**Figure 6:**
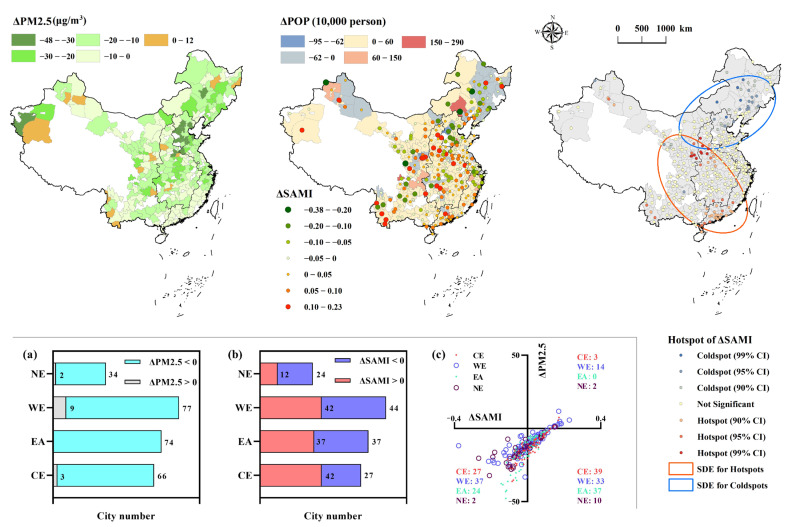
The spatial details of ΔPM2.5, ΔPOP, and ΔSAMI (with its corresponding hotspot and SDE analysis results). The summarized city numbers of (**a**) ΔPM2.5, (**b**) ΔSAMI, and (**c**) quadrantal diagram (ΔPM2.5 and ΔSAMI) with four economic zones (colored number).

In terms of temporal variation, the case cities’ ΔSAMIs did not exhibit similar spatial patterns to their cross-sectional values, even though most cities (251 of 265 cities) showed obvious decline in annual PM2.5 concentration (Figure 6). The city clusters with higher SAMIs showed clear southward shifts judging from the year-to-year morphology changes of SDEs (Figure 4), which was verified by the slight rising trend of ΔSAMI with lower latitudes (Figure 5). Furthermore, Hotspot analysis clarified that the key cities of ΔSAMIs mainly concentrated in CE (ΔSAMI > 0), EA (>0), and NE (<0). Among the four economic zones, NE showed the most significant average variation of −0.04 ± 0.09, followed by CE (0.02 ± 0.07), EA (−0.01 ± 0.06), and WE (nearly 0 ± 0.1). In terms of city number, zonal statistics showed that a total of 133 case cities (nearly 50% of the total cities) experienced increasing SAMIs, with 42 in CE (61%), 37 in EA (50%), 42 in WE (49%), and 12 in NE (33%). There were 119 out of 133 cities with positive ΔSAMIs that were experiencing reduced PM2.5, while another 90 cities showed declining characteristics in both PM2.5 and SAMIs (Figure 6c).

**Figure 7:**
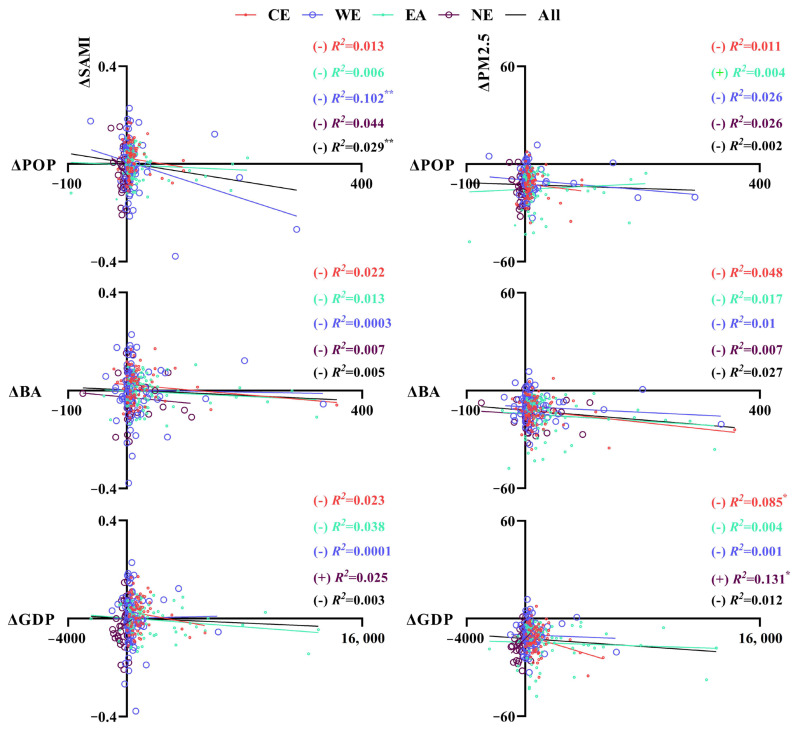
The pairwise linear regression relationships between ΔSAMI/ΔPM2.5 (μg/m^3^) and ΔPOP (10,000 person)/ΔBA (km^2^)/ΔGDP (100 million RMB) in different zones. For all of the case cities; (+)/(−) indicate a positive/negative pairwise relationship; **: *p* < 0.01; *: *p* < 0.05.

Figure 7 shows the linear relationships between ΔSAMI and urbanization indictors in different zones, with an additional analysis on ΔPM2.5. Generally, the results showed that incremental urbanization would lead to declining trends, both in ΔSAMI and ΔPM2.5, in most zoning contexts. Except for the cases between ΔSAMI and ΔGDP in NE, ΔPM2.5 and ΔPOP in EA, ΔPM2.5, and ΔGDP in NE showed positive relationships. However, all these indicators were not closely correlated, with insignificant (*p* > 0.05) or unsatisfactory (lower *R*^2^) regression performances.

## 4. Discussion

### 4.1. Revisiting Urban PM2.5 Pollution in the Perspective of Scaling Law

As mentioned above, the Chinese government implemented a series of strict, costless, and nationwide top-down policies for air pollution abatement in recent years [17,38]. Our study revealed remarkable improvement in urban PM2.5 pollution levels across most cities in China after a brief changing analysis on its annual concentration (2015–2019) (Figure 6). In contrast to previous studies, prior to 2013–2015, which documented that the overall risk of air pollution in China was increasing [48,54,55], our findings suggested that current environmental policies are functioning effectively. Similar conclusions have also been drawn in related studies [33,56,57].

**Figure 8:**
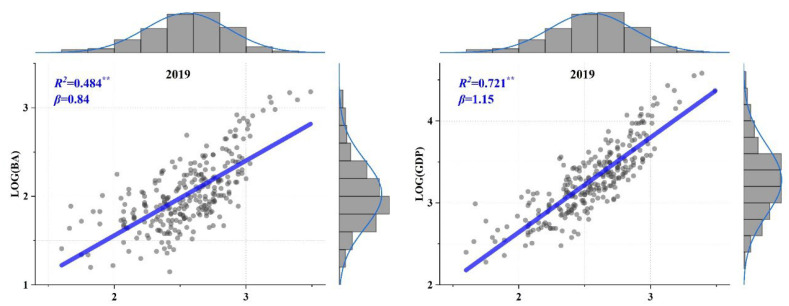
Case example of the fitted urban scaling law for POP and urban built-up area (BA, km^2^) and gross domestic product (GDP, 100 million RMB) in 2019. The straight line refers to the expected linear relationship between these logarithmic indicators for the case cities. **: *p* < 0.01.

However, when we shifted our perspective and tried to discuss this air pollution issue in terms of scaling law, we ended up with interesting new findings. Previously, urban scaling law has been determined globally to be an effective universal taxonomy in quantifying various urbanized characteristics assigned with population sizes [8,12,49]. Commensurate results could also be obtained based on the data used in this study (by comparing Figure 1 and Figure 8), which informed the general law that urbanization is a densification process of resources, capital, and population. The efficient utilization of urban resources will inevitably lead to faster growth of urban virtual factors, such as GDP or technological innovation (weighted by population size, *β* > 1), compared to that of urban infrastructure (e.g., BA or road network, *β* < 1) [7,40]. In this study, the annual PM2.5 concentration, although not a common-sense representative urban indicator, showed a significant sublinear scaling law relationship with urban (population) size and much lower *β* than other types of urban indicators (Figure 1 and Figure 3). From this point of view, this finding also confirmed the effectiveness of environmental policies on air quality improvement, i.e., the air pollution level has significantly lagged behind urban growth.

**Figure 9:**
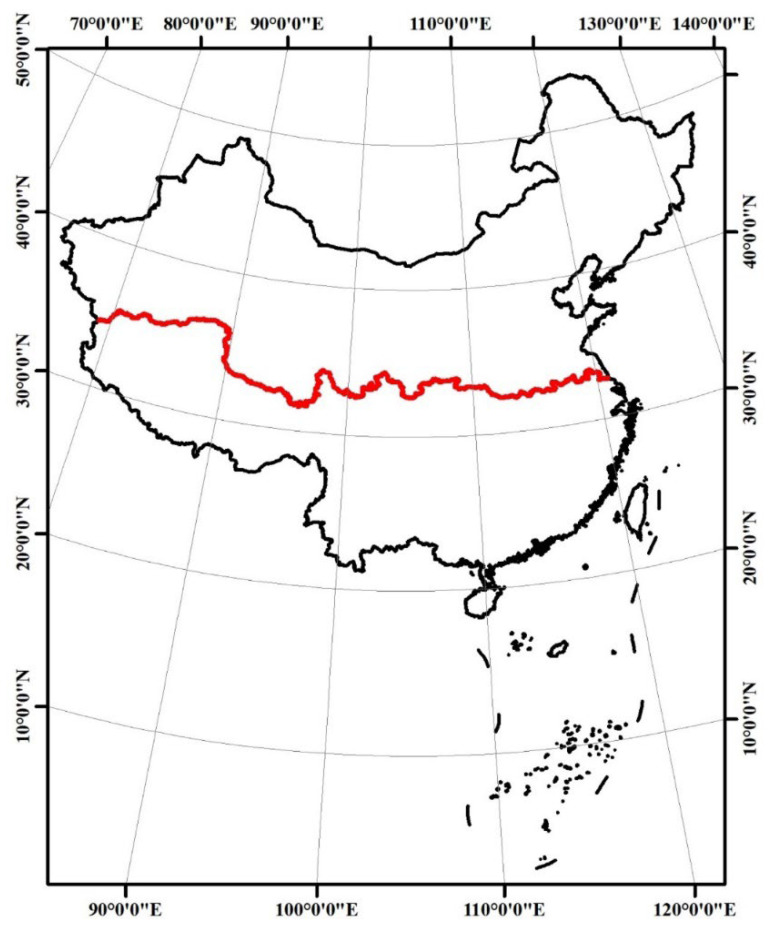
The range of the Qinling Mountain–Huai River winter heating policy (red) line in China, referring to Ebenstein, Fan [18].

However, *β* only indicated global-level scaling characteristics [11,50], whereas SAMI analysis revealed more spatial details. Geographically, higher SAMI can characterize more severe human–land conflict in urban systems in terms of urbanization and air pollution to a certain extent. Thus, our findings indicated that those cities with higher SAMIs have not reached the national average level in terms of coordinating urban development and environment pollution. In other words, in these cities, there was not enough potential space for PM2.5 emissions under the current population size. Thus, future population growth would easily exacerbate this human–land conflict. Generally, in the broader context of national air environmental improvement, our study showed significant heterogeneity in SAMIs across time and space. The cities with higher-ranked SAMIs were mainly distributed in parts of central and western China, while those with lower SAMIs were mainly located in the southern coastal regions. However, the spatial pattern of SAMIs was not consistent with the size of each city; rather, it was related to the current PM2.5 pollution level (Figure 4). To some extent, this can be explained as influenced by the unique natural condition, production, and living styles of different regions, and the cities with serious human–land conflicts are often the areas with serious pollution [26,58]. For example, cities in CE (which showed the highest average SAMI than other regions, Figure 5) had relatively high population sizes and were definitely the most polluted areas identified by many relevant studies, whereas higher PM2.5 pollution risks, weighted by population, were mainly contributed by the local energy-intensive pillar industries [55,59,60,61]. In contrast, as the frontier and autonomous minority regions, cities with small populations in WE also showed higher concentrations of SAMIs and PM2.5 pollution, mainly due to the facilitation of local natural sources (proximity to the Taklamakan Desert) and climatic transport conditions (windy & arid) [41,62]. Moreover, we found quadratic polynomial distribution patterns of SAMIs along the latitude, with high values are around 35°N (Qinling Mountain–Huai River) during 2015–2019, which coincided with the division of north and south China. Ebenstein, Fan [18] argued that the Huai River winter heating range (Figure 9) produced sustained differences in airborne PM concentrations between the north and south of China. This could also partially explain the spatial differences in air pollution and the SAMIs of this study.

**Figure A1:**
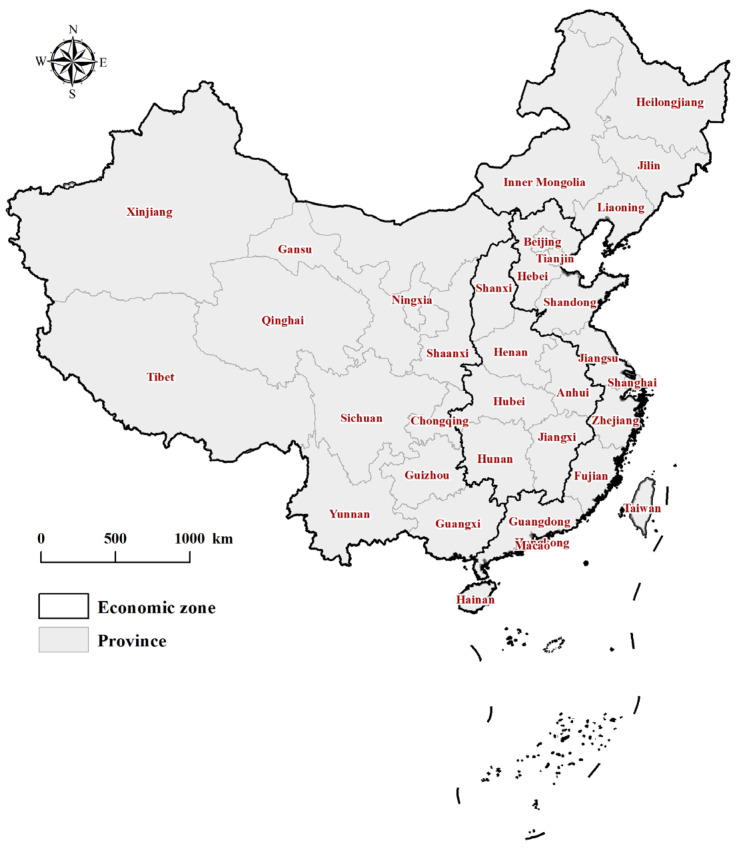
The spatial boundaries and label names of Chinese provinces.

From the temporal change, nearly half of the case cities (133/265) showed rising levels of SAMIs, while nearly all the case cities saw their annual PM2.5 concentration fall (Figure 6). However, this did not mean that the pollution risks in these cities were increasing, but rather emphasized the contradiction between their urbanization process and environmental degradation, which increased relatively compared to the global average status. Moreover, the variation of SAMIs in the case cities did not show clearly regional cluster characteristics in spaces similar to their cross-sectional values. Hotspot and SDE analysis allowed us to note the presence of north-cold and south-hot patterns, in terms of changes, during 2015–2019, which was exactly the opposite of the north-hot and south-cold patterns assigned to the cross-sectional data. The hotspots of ΔSAMI concentrated in central and southern cities. For the cities in central regions (mainly in Henan and Shanxi provinces, Appendix A Figure A1), the relatively high ΔSAMI of these cities was possibly due to the increased relative risk of PM2.5 pollution compared to the surrounding region’s stronger environmental policies (i.e., the Beijing–Tianjin–Hebei (BTH) region, known as the most polluted region in China, Figure 4). Figure 6 shows that the BTH region exhibited the highest reduction in annual PM2.5 concentrations in China. Dong and Wang [56] reported that the source contribution of PM2.5 in the BTH region was skewed from local emissions due to importing from surrounding provinces with less strict pollutant emission control strategies. The above arguments also explained the cities in the BTH region having become the cold spots of ΔSAMI. The cities in the southern region (Guangdong) had less air pollution background (Figure 4) and higher costs for environmental improvement. Scholars have made a rough estimate that reduction of PM2.5 by 1 μg/m^3^ would cost 2.72 million RMB in the BTH region, whereas in the Pearl River Delta (PRD) region (Guangdong province), the cost would reach 7.31 million RMB, because of the need to sacrifice higher value-added industries [63]. This contributed to the insignificant improvement in local air quality (Figure 6), and consequently caused rising SAMIs with increasing risk potential. Recent studies have also suggested the urgent need for synergistic air pollution control in these often-neglected regions of southern China [37,64].

### 4.2. Does Urbanization Still Matter?

**Figure A2:**
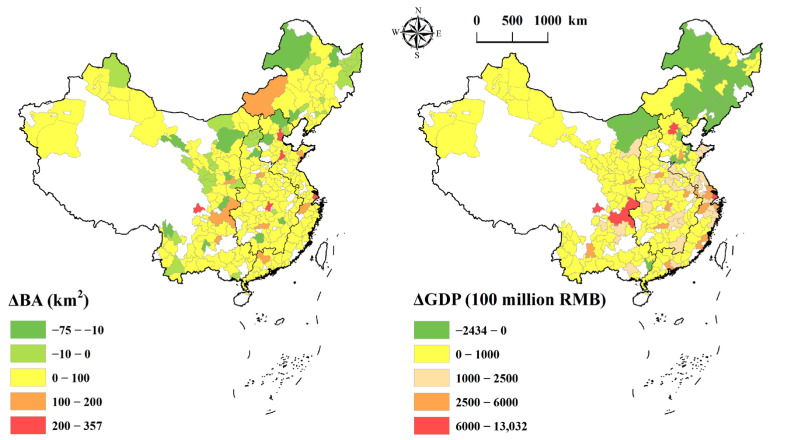
The spatial details of ΔBA and ΔGDP of the case cities.

The process of urbanization has been acknowledged as key in the exacerbation of urban PM2.5 pollution in China, confirmed by dense previous analyses [34,48,65,66,67,68]. However, with the promotion of pragmatic environmental policies and relevant supporting measures, dramatic improvements in domestic air quality have been achieved in a few short years [17,38]. Similarly, significant decreases in annual PM2.5 concentration accompanying urbanization progress have been documented [33]. The question then becomes whether urbanization remains responsible for PM2.5 pollution? The Environmental Kuznets Curve (EKC) hypothesis can usually be used to answer this question, suggesting that urban development would eventually alleviate the environmental degradation (e.g., urban PM2.5 pollution) [69,70,71]. However, our results did not support this hypothesis, as shown in Figure 7, with insignificant pairwise associations between the variation of PM2.5 and urbanization indicators in most of the zoning cases. Notably, NE was an exception, showing a significant positive relationship between ΔPM2.5 and ΔGDP (*p* < 0.05), and implied that the declining GDP in NE (due to the continuous loss of population and socio-economic resources in recent years, Figure 6 and Appendix A Figure A2) was effectively associated with a local reduction in PM2.5 concentration. But this also contradicted the growth progress of urbanization highlighted by the EKC hypothesis [72,73].

SAMIs of the case cities were spatially inconsistent with their urbanization indicators, i.e., POP, BA, and GDP (Figure 4 and Figure A2). In addition, the regression analysis in Figure 7 emphasized that the variations of urbanization indicators were not significantly associated with local ΔSAMIs across different zonings, indicating that the urbanization process might not be the main factor affecting the human–land conflict in terms of urban air pollution.

**Figure 10:**
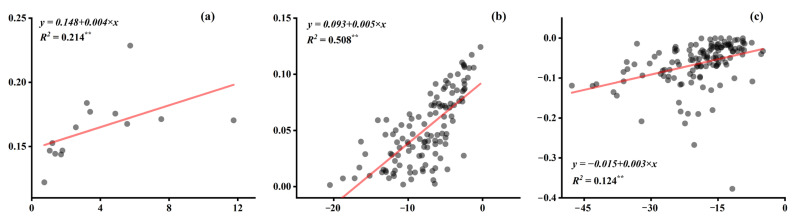
The linear regression relationships between ΔPM2.5 (*x*-axis, μg/m^3^) and ΔSAMI (*y*-axis) in different quadrants (Figure 6c), as (**a**) ΔSAMI > 0 & ΔPM2.5 > 0, (**b**) ΔSAMI > 0 & ΔPM2.5 < 0, (**c**) ΔSAMI < 0 & ΔPM2.5 < 0; **: *p* < 0.01.

Did ΔSAMI correlate with ΔPM2.5? Figure 6c showed a rough linear nexus between the two indices. We broke down their relationship in a piecewise fashion according to the quadrant range. All three valid quadrants showed significant (*p* < 0.01) and positive linear relationships between ΔPM2.5 and ΔSAMI (Figure 10). However, in a way, these results contradicted each other: that is, positive ΔPM2.5 undoubtedly increased the local risk in terms of higher SAMIs (Figure 10a), whereas negative ΔPM2.5 not only reduced the risk in some cities (Figure 10c), but also increased the risk in others (Figure 10b). This contradiction further highlighted the inherent heterogeneities in PM2.5 pollution risks in different cities, which cannot be simply summarized by socioeconomic development or environmental improvement. Similarly, Zhao, Zhou [53] concluded that different cities would show different gradations of PM2.5 pollution characteristics according to their urban size (BA), landscape form, and spatial location; Wang, Yao [33] also reported that demographic/industrial structures in different cities could also contribute to differences in the core drivers of local PM2.5 pollution risks—not to mention the direct impact of natural factors with distinct geographical characteristics, e.g., climate, topography, and land cover, etc., on the spatial heterogeneities of air pollution risk [30,41,74,75]. Under the framework of urban scaling law, this study revealed that the cities in high-value clusters of ΔSAMI were not commonly thought of as economic-developed mega cities with a greater spotlight (i.e., Beijing and Shanghai) or as underdeveloped small cities. Instead, they were primarily medium-sized cities located in CE and EA (Figure 6 and Figure A2), which presented relatively intensified pollution risk potential along with their urbanization process but have not received sufficient attention in previous PM2.5 related studies [76,77,78].

### 4.3. Implications and Limitations

To our best knowledge, this study was the first attempt to discuss the urban scaling law characteristics of PM2.5 pollution, providing a brand-new perspective on the quantitative nexus between the process of urbanization and its related environmental risks. In general, the significant sublinear relationship (*β* < 1, *p* < 0.01) between PM2.5 and POP objectively affirmed the unremitting effort on the part of the Chinese government to improve nationwide air quality. However, all was not well; SAMI analysis further revealed significant regional gaps in terms of relative risk. This demonstrated that environmental policies implemented alongside urbanization processes effectively mitigated the overall air pollution risk but failed to take regional balance into account. From the static cross-sectional perspective, the relative risks of the case cities exhibited a distinct spatial gradient with higher clusters in CE and lower clusters in other economic zones. Its spatial pattern was broadly consistent with that of PM2.5 pollution, implying that the current focus on environmental investment needs to be maintained, especially for those cities with higher PM2.5 concentrations than the ambient air quality standard of II level (35 μg/m^3^) in China (Figure 4). More importantly, from the interannual variation perspective, the overall southward trend of higher SAMIs will then contribute to identifying future environmental concerns. Another implication of the study is that relying on urban development alone cannot effectively solve the persistent environmental risks originating from the process of urbanization. Not only that, but the short-term advantage of policies for air quality improvement also come at great urbanization costs, leading to economic slow-down or recession (Figure A2) and subsequent possible population loss (Figure 6), unemployment, or social injustice [39,71,79,80]. Therefore, in the long-term, more sustainable urban environmental solutions with more feasible choices (e.g., cleaner energy and technology, public awareness and participation, regional transfer payments, etc.) are essential for narrowing the relative risk gaps of PM2.5 pollution across cities, independent of their urbanization levels [16,27,81].

However, this study had some limitations. First, it is necessary to note the lower model performance (*R*^2^) of the annual PM2.5 concentration (Figure 3) compared to other types of urban indicators in previous urban scaling studies (Figure 1). This suggested that environmental degradations were not inherent characteristics along with urban development and may show significant regional and temporal heterogeneity. Zoning analysis verified this, exhibiting various properties in both PM2.5 pollution and related SAMIs among CE, EA, NE, and WE (Figure 6). In addition, analysis using global statistical analysis algorithm (Equation (1)) in such region with significant spatial heterogeneity will result in lower *R*^2^ due to the spatial non-stationarity effect [82]. Similarly, Zhao, Zhou [83] obtained lower *R*^2^ (< 0.1) when they discussed the regression relationship between the PM2.5 concentration and urban population across the Chinese cities. Thus, it will be essential to carry out more detailed studies to discuss the potential heterogeneity of the scaling law for more diverse regions in the future. Second, the urbanization factors (i.e., POP, BA, and GDP) did not show significant association with SAMIs of the case cities, and further research is needed to explore more relevant explanatory variables to indicate the underlying causes or driving mechanisms of the variations in SAMIs.

## 5. Conclusions

This study examined the potential urban scaling law that may lie in the urban air pollution concerns of the public in China, using 265 cities as case study. Based on the ground-monitored PM2.5 concentration and statistical socioeconomic data during 2015–2019, we carried out a series of analyses and discussions. The main findings were summarized as follows:

(1) Significant scaling law was examined between PM2.5 pollution and urban population size, and their sublinear relationship indicated that air quality degradation significantly lagged behind urban growth, affirming the remarkable effectiveness of national efforts on atmospheric environment improvement during the study period.

(2) SAMIs reflected the relative conflict risk between PM2.5 pollution and urbanization and showed significant regional cluster characteristics. Cities in CE generally showed a higher risk than other regions, but there was a clear southward tendency for the city clusters with positive ΔSAMI from 2015 to 2019.

(3) Neither urban scales nor temporal variations showed significant association with POP, BA, or GDP, indicating that the urbanization process was not the main factor affecting the human–land conflict on the dynamic of urban air pollution after the implementation of the national environmental policy in China.

The aim of this study was to extend the effectiveness of the urban scaling law onto a wider topic by innovative incorporation of the scaling model into urban environmental risk study. The work offered a new perspective from which to reframe the urban PM2.5 pollution risk along with the nationwide air environmental effort in China. Both the methodology and findings of this study will be beneficial for future efforts on similar urban environmental degradations, such as urban overheating, flooding, or water pollution issues.

## Data Availability

The data presented in this study are available on request from the corresponding author.
